# A Pound of Flesh: What Cachexia Is and What It Is Not

**DOI:** 10.3390/diagnostics11010116

**Published:** 2021-01-12

**Authors:** Emanuele Berardi, Luca Madaro, Biliana Lozanoska-Ochser, Sergio Adamo, Lieven Thorrez, Marina Bouche, Dario Coletti

**Affiliations:** 1 Department of Development and Regeneration, KU Leuven Campus Kulak, 8500 Kortrijk, Belgium; emanuele.berardi@kuleuven.be (E.B.); lieven.thorrez@kuleuven.be (L.T.); 2 Faculty of Rehabilitation Sciences, REVAL, Hasselt University (UHasselt), 3590 Diepenbeek, Belgium; 3 DAHFMO Unit of Histology and Medical Embryology, Sapienza University of Rome, 00161 Rome, Italy; luca.madaro@uniroma1.it (L.M.); biliana.lozanoska-ochser@uniroma1.it (B.L.-O.); sergio.adamo@uniroma1.it (S.A.); dario.coletti@uniroma1.it (D.C.); 4 Biological Adaptation and Ageing, CNRS UMR 8256, Inserm U1164, Institut de Biologie Paris-Seine, Sorbonne Université, 75006 Paris, France

**Keywords:** cachexia syndrome, diagnosis, biomarkers, muscle wasting, 3D skeletal muscle models, chronic degenerative diseases

## Abstract

Body weight loss, mostly due to the wasting of skeletal muscle and adipose tissue, is the hallmark of the so-called cachexia syndrome. Cachexia is associated with several acute and chronic disease states such as cancer, chronic obstructive pulmonary disease (COPD), heart and kidney failure, and acquired and autoimmune diseases and also pharmacological treatments such as chemotherapy. The clinical relevance of cachexia and its impact on patients’ quality of life has been neglected for decades. Only recently did the international community agree upon a definition of the term cachexia, and we are still awaiting the standardization of markers and tests for the diagnosis and staging of cancer-related cachexia. In this review, we discuss cachexia, considering the evolving use of the term for diagnostic purposes and the implications it has for clinical biomarkers, to provide a comprehensive overview of its biology and clinical management. Advances and tools developed so far for the in vitro testing of cachexia and drug screening will be described. We will also evaluate the nomenclature of different forms of muscle wasting and degeneration and discuss features that distinguish cachexia from other forms of muscle wasting in the context of different conditions.

## 1. Introduction

Cachexia is a multifactorial syndrome characterized by body weight loss, declining muscle mass and function, wasting, and inflammation of adipose tissues accompanied by metabolic disarrangement, anorexia, systemic inflammation, and insulin resistance. Cachexia is typically associated with a number of underlying chronic degenerative diseases, such as cancer, chronic obstructive pulmonary disease (COPD), HIV, chronic heart failure, diabetes, liver failure, rheumatoid arthritis, and chronic kidney failure [1]. Here, we will chiefly refer to cancer-associated cachexia as a representative and widespread, albeit not the most common, form of cachexia. Of note, cachexia is also associated with acute states (see below) and, in this case, may last much longer than the causative event itself.

The clinical relevance of cachexia is shown by its impact on both the prognosis and the efficacy of treatment against the underlying disease as well as survival time and quality of life. Yet, the importance of this syndrome is often overlooked by healthcare professionals. The lack of commonly agreed-upon diagnostic criteria for cachexia, and the poor attention given to the nutritional status of the patients, have made it difficult to establish efficient clinical management of this syndrome, which remains largely obscured by the underlying diseases.

A key aspect of the growing interest in the clinical management of cachexia has been the need for its accurate definition. Although a consensus definition for cachexia has been reached, guidelines for its diagnosis and clinical management are still poorly diffused. Considering that up to 80% of cancer patients develop cachexia, and at least 20% of cancer-related deaths per year [2,3] are directly attributed to this syndrome, there has been a strong need to reach a consensus on the definition of cachexia in order to improve the clinical management of cancer patients.

This review aims to provide the state of the art of cachexia diagnosis, its definition in relation to different primary chronic diseases, and its assessment criteria. Thus, it offers a tool for healthcare professionals to establish diagnostic criteria and guidance for the clinical management of cachexia.

## 2. The Evolving Concept of Cachexia

Cachexia is a multifactorial syndrome occurring during both acute and persistent catabolic events such as malnutrition and chronic degenerative diseases. The concept of “chronic degeneration” can create confusion when associated with a complex syndrome like cachexia. By definition, chronic degeneration determines the progressive loss of function or deterioration of a tissue or organ in the absence of a single causing event (e.g., an acute trauma) and is responsible for premature disability, mortality, and morbidity. This picture highlights the clinical relevance of cachexia. Nevertheless, the progression of degeneration may be faster or slower in absolute terms. In healthy aging, for example (without the presence of any disease), these processes are significantly slower and occur significantly later than those observed in degenerative diseases. By contrast, cachexia arising from acute events (i.e., nondegenerative events in the long term), such as trauma, burn, acute infection, or toxicity, can be reversed by therapy interventions aimed at the primary cause of cachexia, even though the signs of cachexia often last for a long time following the recovery of the patient.

Indeed, cachexia is associated with a plethora of chronic diseases, which, for their nature, can be all considered degenerative diseases, including cancer, organ failure (COPD), heart or kidney failure, infectious (AIDS), autoimmune (rheumatoid arthritis), metabolic (diabetes) diseases, etc. All these conditions share several underlying mechanisms and ultimately have a similar output, i.e., severe muscle wasting. However, the multifactorial origin of cachexia and its multisystem involvement made the study of this syndrome in the context of underlying chronic diseases complicated and, consequently, difficult to develop a definition of cachexia [4]. Therefore, the current definition of cachexia is the result of a prolonged effort and continuous evolution; the major advances toward a definition of cachexia, which would be both accurate at the molecular level and clinically exploitable are summarized in Table 1.

In 2008, cachexia was defined as a “complex metabolic syndrome associated with underlying illness and characterized by loss of muscle with or without loss of fat mass” [5]. According to this definition, weight loss is the main feature of cachexia. In particular, the assessment criteria included a minimum of 5% of weight loss during the last 12 months (or a BMI < 20.0 kg/m^2^ whenever a history of weight loss cannot be documented) in the presence of underlying illness and three of the following features: decreased muscle strength, increased fatigue, anorexia, low fat-free mass index, abnormal biochemistry, increased inflammatory markers, anemia, and low serum albumin [5]. This definition distinguishes cachexia from starvation, malabsorption, age-related muscle mass loss, and hyperthyroidism, representing the first attempt to define a condition that was still often misleadingly referred to as “cachexia/anorexia syndrome [5]”.

Indeed, the evolution of the concept of cachexia is related to the nutritional status of the patient and the clinical outcome of the underlying disease. Deterioration in the nutritional status of the patient is a common consequence of chronic degenerative diseases and worsens the prognosis [4]. On the other hand, forced nutrition distinguishes anorexia from cachexia since the latter cannot be counteracted by enteral or parenteral nutrition [6]; thus, anorexia exacerbates, but does not coincide with, cachexia. In healthy subjects, metabolic disturbances arising from an intermittent or even prolonged period of starvation can be easily reversed without inducing permanent catabolic events involving skeletal muscle tissue. Conversely, in disease-associated malnutrition (which is also named cachexia, see below), reduced intestinal absorption and malnutrition (i.e., asthenia, anorexia, early satiety, nausea) contribute to progressive body weight loss and muscle wasting [4]. The reduced energy intake triggers catabolic adaptive responses, which induce proteolysis and lipolysis in order to compensate for the reduced food intake, further worsening the muscle decay initiated by the high inflammatory background of the underlying chronic illness. Therefore, the involuntary loss of body weight and muscle mass are the most common features of cachexia and are considered diagnostic factors of this syndrome.

Since body weight loss is the main feature of malnutrition, and systemic inflammation is present in the vast majority of the patients affected by chronic degenerative diseases [7], a unified definition for the clinical management of cachexia based on the nutritional status, as well as on the inflammatory background of the patients, was proposed in 2010. Based on these criteria, Jensen and colleagues [7] proposed the following nomenclature: (1) starvation-related malnutrition without inflammation, (2) chronic disease-related malnutrition with a mild to moderate degree of chronic inflammation, and (3) acute disease or injury-related malnutrition with severe inflammation. Nevertheless, the definition of cachexia as disease-associated malnutrition was introduced to unify the concepts of malnutrition-mediated by starvation and malnutrition-mediated by inflammation [4].

In 2010, the Special Interest Groups (SIG) [1] also introduced the concept of precachexia based on the following assessment criteria: (1) patients with a chronic disease, (2) small weight loss (≥5% of usual body weight during the last six months), (3) a chronic or recurrent systemic inflammatory response (evaluated by the serum levels of inflammatory markers like C-reactive protein [8,9]), and (4) anorexia [1].

In 2011, a new definition of cancer-related cachexia was proposed [10], building on the previous definition released for “general” cachexia in 2008 [5]. According to this new definition [10], which also takes into account the nutritional status of the patients, cachexia is a “multifactorial syndrome defined by an ongoing loss of skeletal muscle mass (with or without loss of fat mass) that cannot be fully reversed by conventional nutritional support and leads to progressive functional impairment” [10]. Based on this definition, the abnormalities associated with cachexia stem from a combination of altered metabolism and reduced food intake in cancer patients, leading to a negative protein and energy balance. Moreover, other important advances from the international consensus on cancer-related cachexia released in 2011 included new diagnostic criteria (more than 5% of weight loss over the past six months in the absence of simple starvation or BMI < 20 or weight loss > 2%) and updated the staging of the syndrome, outlined by three consecutive clinical stages: precachexia to cachexia to refractory cachexia [10].

Based on the paradoxical observation that obese people are protected from cachexia-associated morbidity [11], the medical community realized that taking into account BMI alone was not able to mirror the severity of cachexia and that the body weight loss (with respect to the initial weight) had to be taken into account, instead. For this reason, Baracos and collaborators proposed an additional bivariate classification that defined five stages of cachexia based on a combination of BMI and weight loss [12] that has been recently validated in clinical practice [13].

Overall, the efforts to reach an accurate and clinically-sound definition of cachexia are important for several reasons: (1) to heighten the awareness of conditions potentially inducing cachexia; (2) to improve the staging of cachexia, thus helping in the identification of novel diagnostic/prognostic markers; and to (3) speed up the diagnosis of cachexia. Each “new” definition of cachexia presented in Table 1 amended the previous definition, ameliorating and, somehow, replacing it. These guidelines definitely helped to deal with the diagnosis and management of cachexia. An important issue that still awaits clarification is the relationship between cachexia and malnutrition [1]. Indeed, while not all malnourished patients are cachectic, all cachectic patients are invariably malnourished [1].

## 3. Use and Misuse of the Term “Cachexia” in Fasting, Muscle Disuse and Sarcopenia

The biology behind the muscle mass loss observed in different catabolic conditions determines the need to adopt stringent definitions and appropriate terminology for several conditions that are similar, insomuch as they share muscle wasting. Cachexia, for example, is characterized by the progressive wasting of lean body mass and adipose tissue, mainly mediated by inflammation, while, during fasting, the reduced intake of nutrients is what leads to increased lipolysis without a significant loss of muscle proteins. This is why nutritional supplements alone turned out to be mostly ineffective in restoring skeletal muscle mass and preserving body wasting, although they may promote fat mass deposition [14].

**Figure 1:**
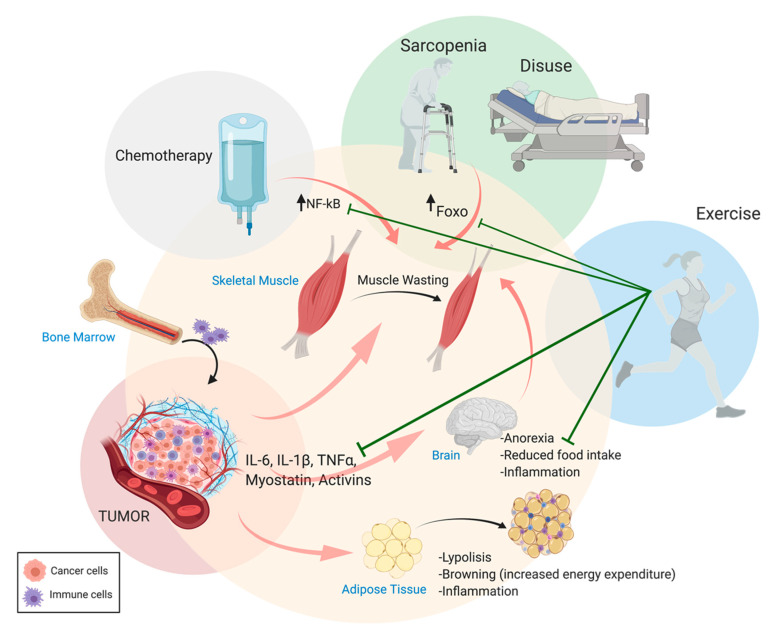
Systemic catabolic crosstalk in cachexia. Proinflammatory cytokines produced by immune and tumor cells (i.e., in cancer-related cachexia) and other circulating molecules trigger catabolic events in skeletal muscle tissue, adipose tissue, and the central nervous system (CNS). Chemotherapy, sarcopenia, and disuse aggravate cachexia outcomes during physical activity ameliorates prognosis by counteracting muscle wasting and the inflammatory condition. Figure made using BioRender.com.

Skeletal muscle mass accounts for 60% of the body’s protein store [15] that can be easily mobilized to provide the liver and immune system with amino acids in the case of metabolic stress [16]. Muscle atrophy occurs when the overall rate of protein degradation (importantly affecting contractile proteins) exceeds the rate of protein synthesis, leading to an imbalance in favor of catabolism [17,18]. Indeed, during cachexia, the selective degradation of skeletal muscle proteins, through the activation of ubiquitin–proteasome machinery, leads to the degradation of sarcomeric proteins as seen in other catabolic conditions, including muscle disuse [17,18]. Interestingly, cancer and resting conditions seem to be regulated by different signaling pathways driving the degradation of distinct sarcomeric proteins, and a combination of disuse and cachexia may result in cumulative adverse effects driving muscle wasting [19]. Cachexia leads to reduced mobility due to fatigue, thus muscle wasting is further exacerbated by disuse in conditions such as cancer cachexia. It is now well established that muscle activity (i.e., physical activity and exercise training) protects against cancer-related muscle wasting and promotes the maintenance of muscle mass [20,21,22,23,24] (Figure 1). A particular form of disuse is neurogenic muscle atrophy, which shows both common and divergent pathways with cancer cachexia [25].

Sarcopenia is characterized by a chronic deficit in muscle protein storage, which results in the progressive loss of muscle mass quality and strength and/or physical performance [1,26,27,28]. So far, two distinct forms of sarcopenia have been described: age-mediated loss of muscle mass (primary sarcopenia) and loss of muscle mass without the emphasis on muscle function (secondary sarcopenia or disease-related sarcopenia), which can be associated, among others, with COPD, heart, and renal failure [29]. Although sarcopenia is typically observed in the elderly [27,28,30], it can be a severe comorbidity of cancer (secondary sarcopenia), malnutrition, and disuse conditions in young subjects [1]. Nevertheless, most sarcopenic individuals are not cachectic. Signs of sarcopenia have also been observed in subjects that do not show weight loss, anorexia, or measurable systemic inflammatory response. Furthermore, after acute inflammatory stress, sarcopenia may be accelerated and, in the elderly, can also involve a low-grade systemic inflammatory response [31] or insulin resistance [32]. Considering the clinical relevance of sarcopenia, its definition is continuously evolving to improve its diagnosis and clinical management. In 2018, an operational definition of sarcopenia was proposed based on the following criteria: (1) low muscle strength = probable sarcopenia, (2) low muscle quantity or quality = confirmation of diagnosis, and (3) low physical performance. In the presence of Criteria 1–3, sarcopenia is considered severe [26]. In 2019, the task force of the Society for Sarcopenia, Cachexia and Wasting Disorders encouraged health care professionals to screen for sarcopenia using the SARC-F questionnaire [29] consisting of the following components: Strength, Assistance with walking, Rise from a chair, Climb stairs and Falls [33], confirming the pivotal importance of assessing muscle function for the diagnosis of sarcopenia.

Notably, the most recent consensus definitions of different forms of muscle wasting, from aging-associated sarcopenia to cancer cachexia, include the loss of muscle function, which mirrors the importance of the functional status of the musculature and is particularly relevant for the quality of life of the patients [34].

An additional, relevant feature of cachexia is the presence of high levels of proinflammatory cytokines in the bloodstream. These mediators are produced by both the immune system and, in the case of cancer-related cachexia, by the tumor cells (Figure 1). Circulating cytokines promote muscle wasting by activating specific signaling pathways in muscle fibers. Increased circulating levels of proinflammatory cytokines, such as IL-6, IL-1β, TGFβ, and IFN-γ, and decreased anabolic hormone synthesis (i.e., testosterone) have been described in response to tumor growth [35]. Consequently, the activation of the downstream catabolic pathways (i.e., JAK/STAT, SMAD, FOXO, and NF-kB) in muscle fibers triggers muscle protein breakdown [36,37,38,39,40]. Conversely, their inhibition prevents cancer-related cachexia by restoring muscle mass [41,42,43].

Targeting myostatin is known to prevent muscle wasting due to unloading during space flights [44], and proinflammatory cytokines play a role in wasting during reduced muscle activity, such as the recent COVID-19 quarantine [45]. However, the global profile of humoral factors associated with different forms of muscle atrophy is not always the same: for instance, while myostatin is upregulated in both disuse- and cancer-induced muscle atrophy, IGF-1 levels vary in opposite ways [46]. Likewise, recent studies have reported differences in protein metabolism in short-term muscle disuse [47] compared to cancer cachexia [24]. Unfortunately, due to the pleiotropic effects of myostatin, targeting this myokine is not a straightforward approach [48].

## 4. Markers of Cachexia Used in the Clinic

Loss of weight is the main clinical feature contributing to the development of a cachectic phenotype, mostly due to muscle wasting [10,49]. Loss of muscle homeostasis and the switch to a higher rate of protein degradation loop toward the progressive deterioration of muscle function. Reduced muscle strength and increased fatigue impact the quality of life, the health status, and the potential of administered therapies. Diagnostic markers should reflect the above changes. These markers can be grouped into four categories: cytokines (corresponding to “inflammation” in the original consensus definition of cachexia by Evans et al. in 2008 [5]), lean muscle mass (“muscle wasting”), markers of biological activity and metabolism (“altered metabolism”), and other humor factors.

Overall, unintentional weight loss, elevated blood levels of CRP, IL-6, IL-1β, TNFα, and muscle function decline should be assessed in any patient to diagnose cachexia independently from the underlying disease. In particular, reduced appetite, early satiety, nausea, and other abnormal eating behavior should be evaluated together with body composition analysis [4]. Interestingly, cachexia is not simply characterized by increased levels of proinflammatory cytokines, but rather by an increase in the ratio between pro- and anti-inflammatory cytokines [50,51]. As a consequence, the evaluation of serum cytokines should be extended to both categories of these factors. It is worth noting that the source of inflammatory cytokines and other procachectic factors may be the tumor, the immune tissue, the fat tissue, as well as the muscle tissue itself [52,53].

Since muscle wasting plays a crucial role in the pathophysiology of cachexia, a correct diagnosis is necessary by measuring fat-free muscle mass using several methods, including bioimpedance analysis, computed tomography, and a dual-energy X-ray absorptiometry scan. Unfortunately, the costs and availability of these procedures (mainly offered only by large health care institutions) still limit their use [54]. To circumvent these limits, muscle function is currently evaluated by physical performance tests, including total activity, stairs climbing, 6 min walk distance, handgrip strength, and chair sit-to stand [55].

The onset of cachexia can also occur before any evident weight loss and muscle wasting; thus, it is critical to search for specific biomarkers to facilitate early diagnosis and therapy response [56]. So far, numerous affordable, reliable, and easily available serological biomarkers for the early detection of muscle loss have been proposed. Among these, creatinine is a reliable muscle mass biomarker [54]. Under stable kidney function, creatinine is produced at a constant rate by the body depending on the absolute amount of muscle mass [57]. However, analysis of creatinine clearance is crucial to examine renal function, especially in patients with chronic kidney disease [58]. General limitations in the use of creatinine as a biomarker of muscle mass include the high costs of the methods of analyses [54] and the variation induced by meat intake [57]. Moreover, several neoepitopes (i.e., parent proteins produced by post-translational modifications of existing molecules) [54] derived by the degradation of structural and functional proteins in the skeletal muscle tissue have been suggested as biomarkers of muscle wasting. This group includes sarcomeric proteins such as myosin, tropomyosin, troponin and actin, and extracellular matrix proteins such as laminins [59]. Type VI collagen turnover-related peptides were proposed as serological biomarkers for immobilization/remobilization studies, and in particular, type VI collagen N-terminal globular domain epitope (IC6) and MMP-generated degradation fragment of collagen 6 (C6M) were identified as good biomarkers of both muscle mass and muscle function changes in young men [60]. Serum levels of N-terminal propeptide of type III procollagen *(P3NP)* are associated with changes in lean body mass and appendicular skeletal muscle mass. P3NP also plays a role as a predictor of anabolic response to growth hormones and testosterone [61]. 3-Methylhistidine (3MH) has been proposed as a marker of muscle protein breakdown through the post-translational methylation of specific histidine residues in actin and myosin with clinical relevance as a predictor of myofibrillar proteolysis [54], while 3-MH-to-creatinine (3-MH/Crea) and 3-MH-to-estimated glomerular filtration rate (3-MH/eGFR) can support the diagnosis of frailty [62]. Growth differentiation factor-15 (GDF-15) plays an important role in the pathophysiology of cachexia. GDF-15 induces muscle wasting, and its association with chronic muscle-degenerative conditions and clinical outcomes has been established for many chronic degenerative conditions, including COPD [63], as well as many cancers [64] such as prostate, urothelial, renal, melanoma, colorectal, cervical, breast, endometrial, thyroid, and pancreatic cancer [65,66]. Myostatin is a negative regulator of muscle growth and a member of the transforming growth factor (TGF-β) superfamily. Theoretically, it is considered a good candidate for inclusion in the panel of muscle-wasting biomarkers. However, the use of myostatin for the diagnosis and prognosis of cachexia is affected by several issues: in particular, contradictory results regarding the correlation of its high serum levels and muscle wasting were reported in sarcopenic patients due to age and gender differences [67], while decreased serum levels of myostatin were found in patients with lung, colorectal, and medullary thyroid cancer [68,69]. Physical activity [70] and nutritional status [71] are also confounding factors for the adoption of myostatin as a biomarker of cachexia [67]. Activin is another member of the TGF-β superfamily: elevated levels of plasma activin have been proposed as an adverse prognostic factor in cancer patients [72], and its causative role in mediating catabolic responses and atrophy in muscle cells has been recently demonstrated in vitro [52]. Follistatin (FST) is an endogenous inhibitor of myostatin- and activin-mediated muscle wasting that works as a positive regulator of muscle growth. Thus, follistatin, too, was investigated as a potential biomarker of cachexia and, especially, sarcopenia [73]. Follistatin not only binds and neutralizes myostatin but also stimulates myoblast differentiation [67,74]. Irisin is an exercise-induced myokine hormone that may be a useful biomarker of the muscle status in cachexia. Several studies reported decreased levels of serum irisin in patients with diabetes [75], chronic kidney disease [76], and myocardial infarction [77]. Other candidate hormones as potential biomarkers of cachexia are leptin, ghrelin, and obestatin, especially for their biological role in cancer-related muscle wasting [54].

Interestingly, in addition to cytokines and other humoral factors, the plasma lipid profile is altered in cachexia [78]. Since some extracellular vesicles are increased in cachexia [79] and microRNAs are also dysregulated [80], it is likely that additional plasma factors will soon be validated as diagnostic markers of cachexia. For instance, endocrine and paracrine hormones (myokines) packaged into extracellular vesicles and released by muscle fibers during wasting conditions or physical activity offer another important source of biomarkers for cachexia [81].

In summary, a number of confounding factors such as age, gender, nutritional status, and physical activity interfere with the identification of single specific diagnostic biomarkers and predictors of prognosis and therapy response with high sensitivity, specificity, and predictive power [67,82]. Using a panel of complementary biomarkers will help to resolve this issue.

Several recent reviews specifically dedicated to (bio)markers of cachexia are available for an exhaustive list of all the above [83,84,85,86].

## 5. Cachexia often Arises from the Synergism of Two Different, Simultaneous Insults: The Example of Cancer Cachexia Combined with Chemotherapy-Induced Cachexia

While chemotherapy represents one of the primary treatment options for cancer patients, there is a clearly established link between the use of chemotherapeutic agents and muscle wasting (Figure 1). Aside from the known effects of tumor growth on muscle degeneration, chemotherapy may directly influence muscle mass by enhancing proteolysis, leading to muscle weakness [87,88,89,90,91], thereby worsening the overall quality and duration of the patients’ life [89,92,93,94].

Interestingly, cancer-induced and chemotherapy-induced cachexia are characterized by distinct metabolic signatures. Thus, chemotherapy-induced cachexia promotes mitochondrial dysfunctions [95,96,97] independently from those caused by tumor-derived proinflammatory cytokines. Specific metabolite signatures may also represent a promising strategy to distinguish between cancer-induced and chemotherapy-induced cachexia [96]. The existence of different underlying mechanisms, in spite of some shared pathways such as the activation of NF-kB [90], likely accounts for the synergism existing between cancer-induced and chemotherapy-induced cachexia. Considering that most cancer patients are also treated with chemotherapy, a vicious circle takes place in which one form of cachexia is exacerbated by the other.

Because of the positive effects of physical activity in counteracting cancer-related cachexia by reducing muscle proteolysis and autophagic flux, as well as the production of inflammatory cytokines [24,98,99,100], exercise training seems to be a promising strategy to counteract cachexia, even in the presence of chemotherapy (Figure 1). In addition, a number of pharmacological strategies have been proposed for the prevention of chemotherapy-induced muscle wasting. They include the activation of the ghrelin receptor by growth hormone secretagogues (GHS). Specifically, it was demonstrated that GHS regulate calcium homeostasis and antagonize chemotherapy-induced mitochondrial dysfunction [95,97]. Alternatively, the inhibition of the activin receptor 2B signaling (as for the receptor for myostatin) has been shown to prevent loss of muscle mass induced by folfiri or doxorubicin administration [101,102,103,104]. Interestingly, activin receptor 2B inhibition affects the production of inflammatory cytokines and promotes protein synthesis without interfering with the activation of atrogenes [101,103]. It is therefore plausible that, in the near future, a specific test panel will allow distinguishing between different cues inducing cachexia and evaluate the relative contribution of different treatments or conditions to cachexia.

Elucidating the mechanisms behind chemotherapy-induced cachexia can improve both therapy outcomes and the quality of life of patients through the development of (1) novel adjuvant therapeutic strategies to counteract chemotherapy-induced cachexia (e.g., personalized exercise training protocols), (2) discovering new molecular intermediates as potential biomarkers for therapy responses, and (3) developing novel chemotherapeutic approaches that do not interfere with muscle homeostasis.

## 6. Crosstalk between Skeletal Muscle and Other Organs in Cachexia

Interorgan crosstalk in the human body is primarily exerted by metabolic, hormonal, and inflammatory mediators, which are altered in acute or chronic pathological conditions associated with the loss of muscle mass and function. Despite the differences in etiology and pathophysiology, all diseases resulting in cachexia are characterized by profound changes in whole-body composition, which, in turn, results from an organ-specific shift in metabolic and hormonal homeostasis. As a consequence, considerable attention has moved toward the study of adipose tissue [105], gut, liver, the central nervous system, heart, and bone and their interaction with the skeletal muscle system [106]. Skeletal muscle is a major site for glucose and amino acid storage, thus influencing both energy and protein metabolism throughout the body. Any change associated with a lower nutrient intake or an increased metabolic rate, which start to deplete liver glucose and fat tissue, can potentially alter the systemic equilibrium and change skeletal muscle from a storage compartment to an active supplier of energy and amino acids, leading to the decreased production and distribution of myokines and endocrine-related signals [107]. Broad attention has been paid to the metabolic, hormonal, and inflammatory communication between skeletal muscle, fat tissue, and liver in cachectic conditions [106], although less is known about the crosstalk between skeletal muscle and other organs such as gut, brain, heart, and bone.

### 6.1. Gut

Beyond the well-characterized muscle–liver–adipose tissue axis, newly emerging evidence of possible interactions between skeletal muscle and the gut microbiota, the composition of which appears to be strongly influenced by physical activity and general performance status, suggests they may play a role in muscle wasting associated with diabetes, frailty, sarcopenia, and other cachectic conditions [108]. In aging subjects, as well as in patients with anorexia nervosa and in sedentary versus trained, shifts in the microbiota toward a less diversified composition have been correlated with decreased muscle function [109]. A pioneering preclinical study comparing muscle mass and functionality in germ-free mice (lacking gut microbiota) and pathogen-free mice (with microbiota) showed that the absence of microbiota correlates with muscle atrophy and is accompanied by the decreased expression of IGF-1, reduced serum choline, and the downregulation of neuromuscular junction-assembling proteins. Of note, microbiota transplantation could rescue the phenotype, pointing to a direct influence on skeletal muscle [110]. However, due to the high interindividual variability observed in the human microbiota composition, additional controlled studies with larger sample sizes are needed to rule out a causal link [109,111,112]. Additional reports suggest a possible role of the gut microbiota in the regulation of PPARs and the peripheral muscle circadian clock [113,114]. The gut has been reported to also influence muscle loss in cancer-related cachexia due to the chronic inflammatory status underlying this specific condition as well as by directly causing nutrient malabsorption because of barrier dysfunctions or through alterations in ghrelin production and distribution [115].

### 6.2. Brain

Evidence of endocrine crosstalk between muscles and the central nervous system has come mainly from the long-held notion that physical activity positively affects several processes that are important for brain functioning, such as vascularization, hippocampal neurogenesis and neuroplasticity, memory, sleep, and psychological wellbeing (decreasing anxiety and depression) [116]. The majority of these effects have been ascribed to the exercise-induced increase and release of brain-derived neurotrophic factor (BDNF), a phenomenon observed both in murine models and human subjects [117,118]. Skeletal-muscle-released factors, so-called myokines and other metabolites, also play an important role in BDNF activation [119]. A comprehensive understanding of how such regulatory mechanisms might be altered in different cachectic conditions is still missing, but several studies have highlighted how changes in brain mediators responsible for food intake and appetite control can induce anorexia in cancer patients [120]. Nevertheless, nutritional approaches in most of the cases are not effective in preventing or reversing body weight loss in cancer cachexia, pointing to a possible more complex bidirectional relationship between acute/chronic diseases and eating behavior [14,120].

### 6.3. Heart

Cardiac abnormalities associated with increased energy expenditure, proteolysis, and oxidative stress are often observed in cachectic cancer patients and are ultimately responsible for cardiac atrophy and heart failure. In particular, beyond fibrosis, in preclinical murine models of cancer cachexia, alterations have been reported in the myocardium, in the composition of the contractile proteins [121], and innervation [122]. In addition, neuroendocrine cardiac players, such as the brain natriuretic peptide (BNP) and the renin–angiotensin system, are still debated for their possible role in the cardiac degeneration observed in cachexia.

On the other hand, it has been known for decades that skeletal muscle structural and functional alterations are often associated with chronic heart failure (CHF) in noncachectic patients [123]. Muscle wasting not only strongly impairs CHF patients’ quality of life by impacting their daily activities through reduced physical capacity but has been also recently acknowledged as an aggravating comorbidity [124] and an independent predictor of survival [125]. These findings are driving a renewed attention from the scientific and clinical community, and technical advances and increased knowledge about muscle physiology have helped in opening new perspectives and identifying fat tissue as an important mediator in the cardiac–skeletal muscle crosstalk [126,127]. Both inflammation (mediated by circulating factors, such as myokines and adipokines) and oxidative/lipidic metabolic shifts seem to play a pivotal role in CHF-related skeletal muscle and fat wasting [126,127], and novel evidence is emerging about possible targets to prevent and overcome this phenomenon [128,129].

### 6.4. Bone

Because of the increasing life expectancy of the human population, it is of extreme importance to understand how muscle and bones interact and how such interactions are affected in pathological conditions such as sarcopenia and osteoporosis [130]. While the relationship between skeletal muscle and bones has long been considered purely structural and mechanical, this view has changed in recent years due to the extensive efforts spent to investigate both systems. Although sarcopenia and osteoporosis are often associated with several pathological conditions, the hypothesis that communication would happen only through the mechanical load that muscles exert on bones cannot explain why muscle atrophy does not account for all osteoporosis cases, and vice-versa [131], or why fractures heal better if they are covered by muscle flaps [132]. Indeed, experimental evidence suggests that the crosstalk between muscle and bones is mediated by diffusion of secreted factors, and it is now clear that both tissues should be considered endocrine organ systems [131]. Myokines such as myostatin, IL-6, and irisin have been shown to influence both bone formation and resorption [133,134,135,136,137]. On the other hand, osteocalcin signaling improves muscle mass and function and is a main mediator of exercise-induced IL-6 secretion from the muscle [138]. Wnt-3 and TGF-β also influence myocyte differentiation and decrease muscle function via oxidative stress, respectively [139,140,141].

## 7. In Vitro Modeling of Cachexia

Many insights into the development and treatment of cachexia are gained from animal models, particularly rodents [142,143]. The latter have allowed us to validate systemic interventions that are readily translational to clinical practice, such as exercise training [22,24,144], and, at the same time, impossible to reproduce in vitro [23,145,146]. However, molecular pathways in human muscles may differ from those in animals [147,148], and therefore, the development of human muscle models is of importance to study cachexia processes, identify and follow the best biomarkers, and test therapeutic strategies. Traditional monolayer cell culture has been extensively used; however, this cannot reliably capture the phenomena at the tissue level such as myotube maturation, force generation, extracellular matrix remodeling, and capillarization [149]. Three-dimensional skeletal muscle models have emerged and are based on tissue-engineering strategies, which have been developed over the last two decades [150,151,152]. Miniaturization has led to the use of these models for high-content drug screening based on changes in muscle contractility as a response to drug exposure [153,154,155]. The hypertrophic effect of exercise was described in the human muscle model in 2002 [156]. Other influencing factors and markers of cachexia have been studied in this context, such as the paracrine release of IGF1 [157] and changes in the extracellular matrix by increasing the concentration of certain amino acids [158].

Although most advances have been made with myogenic cells, including the C2C12 cell line [150,159,160,161,162] and primary muscle-derived precursors [151,152,163], more recently, vascular cells have also been included to generate constructs that better mimic the in vivo situation [163,164,165,166]. By the inclusion of a vascular network, the capillary density can thus be studied. Decreased capillary density is associated with poor prognoses of cachexia and is influenced by TNFα [167]. TNFα was also shown to inhibit myofiber maturation in tissue-engineered myobundles [168].

A model of skeletal muscle atrophy was described based on the buildup of contractile proteins by electrical stimulation, followed by atrophy induction through length reduction [169]. This resulted in a decrease in the isometric tetanic force, myofiber cross-sectional area, protein synthesis rate, and noncollagenous protein content [169]. Other factors also affecting cachexia such as protein metabolism [161], hypoxia [160], growth hormone [162], or mechanical loading [159,170] can be studied. Although these latter studies were described based on engineered murine tissues, the setup can be translated to human tissues.

In parallel with muscle cell cultures, the modeling of lipolysis by the use of cell cultures of adipocytes treated with putative proatrophic factors has led to the first observations of adipose tissue atrophy in cancer cachexia [171]. Since then, in vitro models have been extensively used to elucidate the mechanisms of white and brown tissue wasting in cachexia [172,173].

One of the most intriguing future developments based on the in vitro modeling of wasting processes is the use of human iPS cells to generate tailored myogenic cells and adipocytes (or virtually any additional type of cells) representative of the patients’ diversity [174]. The latter is one of the frontiers in the field of cachexia and remains totally unexplored.

## 8. Conclusions

In summary, the use of a correct, distinct, and widely recognized definition of cachexia syndrome is central for the identification of a muscle-wasting condition that has been neglected for decades while being responsible for a significant percentage of deaths. Correct diagnosis of cachexia requires the adoption of a rigorous assessment of nutritional status, muscle mass and function evaluation, body composition analysis, and estimation of the patient’s quality of life. The identification of novel specific biomarkers for inclusion into specific diagnostic panels is also of pivotal importance for the early detection, prognosis, and therapy response of specific cachexia-induced muscle-wasting conditions. Moreover, the development of three-dimensional skeletal muscle models for the in vitro modeling of cachexia is a promising strategy to identify novel biomarkers and test therapeutic strategies.

Improved clinical management of cachexia will require the development of novel approaches aimed at reducing the adverse effects of chemotherapy-induced cachexia. Patient-tailored adjuvant treatments, such as exercise protocols and nutritional supplements, represent a new frontier for the treatment of cachexia syndrome.

## Figures and Tables

**Table 1:** The evolving concept of cachexia. Listed here are the criteria for the diagnosis of malnutrition, precachexia, and cachexia, including its severity with (from left to right): the corresponding reference, the definition of the cachectic status, and highlights of the main diagnostic criteria used. Cachexia was initially distinguished from anorexia or malnutrition, and, over the years, the progressive nature of muscle wasting (and its relevance to survival) has been highlighted. Ultimately, a staging was proposed for the severity of cachexia depending on the initial status of the patients.

	Source	Definition	Criteria
Cachexia	Evans W. J. et al., 2008	Complex metabolic syndrome associated with underlying illness and characterized by the loss of muscle with or without the loss of fat mass	Weight loss > 5% in the past 12 months and underlying chronic disease; or BMI < 20 and 3 out of the next 5 criteria: Decreased muscle strength (lowest tertile); fatigue; anorexia; low fat-free mass index; abnormal biochemistry: increased inflammatory markers CRP (>5.0 mg/L), IL-6 (>4.0 pg/mL); anemia (<12 g/dL); low serum albumin (<3.2 g/dL)
Chronic disease-related vs. acute disease/injury-related malnutrition	Jensen G. L. et al., 2010	Malnutrition with chronic mild to moderate and severe inflammation, respectively	Weight loss; inflammatory markers
Precachexia	Muscaritoli M. et al., 2010	Early stage of cachexia	Underlying chronic disease; unintentional weight loss ≤ 5% (if any) of usual body weight during the last 6 months; chronic or recurrent systemic inflammatory response; anorexia or anorexia-related symptoms
Cancer precachexia	Fearon K. et al., 2011	Early stage of cancer cachexia	Weight loss < 5%; anorexia and metabolic change
Cancer cachexia	Fearon K. et al., 2011	Multifactorial syndrome characterized by an ongoing loss of skeletal muscle mass (with or without loss of fat mass) that cannot be fully reversed by conventional nutritional support and leads to progressive functional impairment	Weight loss > 5% over the past 6 months (in absence of simple starvation); or: BMI < 20 and degree of weight loss > 2%; or appendicular skeletal muscle index consistent with sarcopenia (males: <7.26 kg/m^2^; females: <5.45 kg/m^2^) and any degree of weight loss >2%
Severity of cancer cachexia	Martin L. et al., 2015	Bivariate analysis to estimate the severity of weight loss (WL) as a function of initial BMI: five degrees of severity are associated with differential median survival	Severity from grade 0 (BMI > 25 Kg/m^2^, WL < 2.5%) to grade 4 (BMI < 20 Kg/m^2^, WL > 6% or BMI < 22 Kg/m^2^, WL > 11% or BMI < 28 Kg/m^2^, WL > 15% etc.)
